# Serratus muscle stimulation effectively treats notalgia paresthetica caused by long thoracic nerve dysfunction: a case series

**DOI:** 10.1186/1749-7221-4-17

**Published:** 2009-09-22

**Authors:** Charlie K Wang, Alpana Gowda, Meredith Barad, Sean C Mackey, Ian R Carroll

**Affiliations:** 1 Department of Anesthesia, Stanford University School of Medicine, Stanford Systems Neuroscience and Pain Lab, Palo Alto, CA, USA; 2 Department of Anesthesia, Division of Pain Management, Stanford University School of Medicine, Stanford Pain Management Clinic, Redwood City, CA, USA

## Abstract

Currently, notalgia paresthetica (NP) is a poorly-understood condition diagnosed on the basis of pruritus, pain, or both, in the area medial to the scapula and lateral to the thoracic spine. It has been proposed that NP is caused by degenerative changes to the T2-T6 vertebrae, genetic disposition, or nerve entrapment of the posterior rami of spinal nerves arising at T2-T6. Despite considerable research, the etiology of NP remains unclear, and a multitude of different treatment modalities have correspondingly met with varying degrees of success. Here we demonstrate that NP can be caused by long thoracic nerve injury leading to serratus anterior dysfunction, and that electrical muscle stimulation (EMS) of the serratus anterior can successfully and conservatively treat NP. In four cases of NP with known injury to the long thoracic nerve we performed transcutaneous EMS to the serratus anterior in an area far lateral to the site of pain and pruritus, resulting in significant and rapid pain relief. These findings are the first to identify long thoracic nerve injury as a cause for notalgia paresthetica and electrical muscle stimulation of the serratus anterior as a possible treatment, and we discuss the implications of these findings on better diagnosing and treating notalgia paresthetica.

## Background

**Figure 1:**
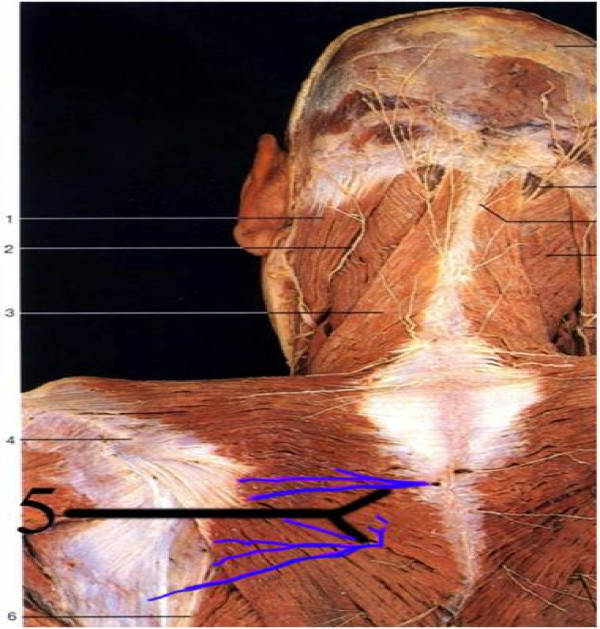
**Medial cutaneous branches of dorsal rami of spinal nerves, drawn in blue; Cutaneous innervations of Notalgia Paresthetica area of presentation, with medial cutaneous branches of dorsal rami of spinal nerves drawn in blue**. Figure adapted from original in Color Atlas of Anatomy by Rohen and Yokochi, 2006.

Notalgia paresthetica (NP) is a poorly-understood condition presenting with pruritus, pain, and paresthesias in an area medial to the scapula and lateral to the thoracic spine. In addition, patients commonly report hyperpigmentation of the skin and other skin abnormalities. It was first described by Astwazaturow in 1934, but both etiology and prevalence of NP are unclear [1,2]. Previous authors have postulated the causes of NP include nerve entrapment of the posterior rami of spinal nerves arising at T2-T6 [3-6], degenerative changes to the corresponding vertebrae [7], and possible involvement of a hereditary component [8]. Cutaneous innervation in this area is provided by the medial cutaneous branches of the dorsal primary rami of the thoracic spinal nerves, which pass through muscles stabilizing the scapula including the rhomboid and trapezius (Figure 1). Immunohistochemical investigations of the symptomatic area have been inconclusive [9-11]. NP has been treated to varying degrees of success with a multitude of palliative approaches directed specifically at the painful or pruritic skin, nerves, and muscle medial to the scapula, including paravertebral nerve blocks [12], cervical epidural steroid injections [13], topical capsaicin [14,15], acupuncture [16], and botulinum toxin type A [17]. Systemic pharmacology used in neuropathic pain more generally has also been directed at NP, including gabapentin [18] and oxcarbazepine [19]. With the exception of a trial of topical capsaicin reporting 30% relief of pruritus [15], no long-acting treatment has shown efficacy in a RCT, and a divergence of explanations for the etiology remains. There is a need to better understand and more effectively treat NP.

**Figure 2:**
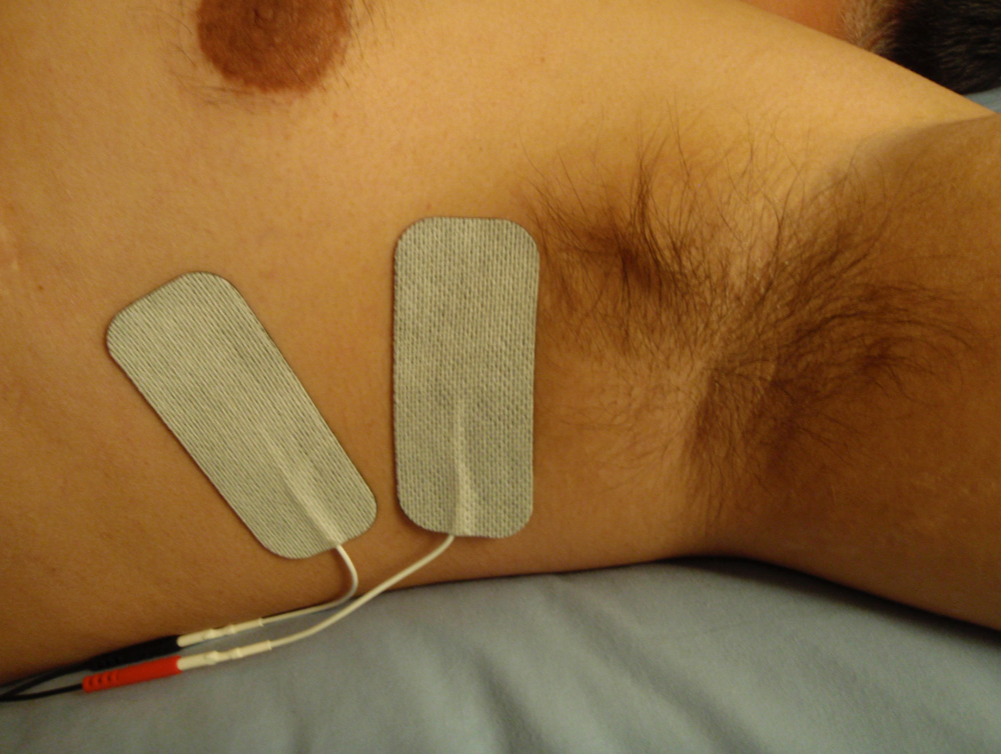
**Transcutaneous electrical muscle stimulation directly to the serratus anterior.** Leads from a home electrical muscle stimulation unit on the lateral side of the scapula, in the axilla and ventral to the lateral border of the latissimus dorsi, providing EMS directly to the serratus anterior and far lateral from the area of pain and pruritus.

The long thoracic nerve arises from branches of cervical nerve roots C5-C7 and innervates the serratus anterior muscle. Injury to the long thoracic nerve or its cervical roots leads to dysfunction of the serratus anterior, with consequent scapular winging or loss of normal scapular protraction. We describe four cases of NP with known injury to the long thoracic nerve or the cervical roots giving rise to the long thoracic nerve, where transcutaneous electrical muscle stimulation (EMS) to the serratus anterior in an area far lateral to the area of pain and pruritus (Figure 2) resulted in significant and rapid pain relief. These findings are the first to identify long thoracic nerve injury with subsequent serratus anterior dysfunction as a cause for NP and EMS of the serratus anterior as a possible successful conservative treatment.

## Case series presentation

All patients reported pain with or without pruritus between the thoracic spine and medial scapula which grew progressively worse throughout the day. Furthermore, all reported exacerbation with activities requiring forward flexion of the arms such as driving, typing, washing dishes, or putting objects on a shelf. Patients did not report skin abnormalities, and no hyperpigmentation was observed. When temperature sensation in the area of maximal pain was tested with ice and compared to the contralateral region, all patients reported reduced sensation of cold without frank numbness. All had injury to the long thoracic nerve or the cervical roots at C5-7 giving rise to the long thoracic nerve, despite the absence of clinically-apparent scapular winging. There was no evidence of hyperpigmentation or other dermatologic symptoms. Prior to treatment with EMS all patients had seen multiple physicians and physical therapists, and tried multiple medications without relief. EMS of the serratus anterior muscle was conducted at 70 Hz with a pulse width of 300 μs to induce tetany of the serratus anterior. Stimulation was prescribed to be 30 seconds on and 30 seconds off for 15 minutes twice a day. All patients reported relief of pain beginning within days upon starting EMS, recurrence of pain if they discontinued stimulation for any prolonged period, and maintenance of analgesic effects with only intermittent stimulation necessary.

The patients described in this case series provided informed consent for the manuscript and/or accompanying images to be published.

### Case 1

A 39-year-old male presented with pain and pruritus medial to the left scapula several months after sustaining an ipsilateral broken clavicle and multiple rib fractures in a bicycle accident. He reported a marked increase in pain with activity utilizing his arms. Unlike his other pain complaints which improved following the accident, over time his left mid-scapular pain became more persistent and refractory. At 60 mg of duloxetine his medial scapular pain was replaced by a sense of itching, and then at 120 mg disappeared completely, although he ultimately could not tolerate duloxetine due to "personality changes". Electrodiagnostic studies revealed cervical root dysfunction at C5, C6, and C7 suspected to be a traction injury from landing on his shoulder after going over the handlebars of his bicycle. The patient began electrical stimulation of the serratus anterior muscle in fall 2007. He reported using the stimulation approximately 15 minutes once a day. He discontinued using it after attaining significant relief only to find that within several weeks the pain reappeared. Upon resuming intermittent stimulation his pain relief returned. By follow-up email in April 2009, he characterized his ongoing relief as "a highly significant improvement in the quality of my life.... Since I started to use [the stimulator] again I have been largely pain free". Treatment with muscle stimulation on an intermittent basis continues after 20 months follow-up.

### Case 2

A 52-year-old male presented with chronic right shoulder and mid-scapular pain and pruritus of 20 years duration, status post ipsilateral thoracotomy and multiple shoulder surgeries, the most recent of which was 2 years previous. The thoracotomy scar ran perpendicularly across the expected path of the long thoracic nerve. He had failed therapy with amytriptyline, valium, skelaxin, fentanyl, dilaudid, methadone, gabapentin, and tizanidine. He reported no intercostal pain, and his notalgia paresthetica scapular pain was located several inches medial to the posterior margin of the thoracotomy scar. He began electrical stimulation of the serratus anterior muscle in July 2008. In a follow up by email in April 2009, he reported pain relief as an "8 on a scale of 10" and continues daily use of the stimulator. Follow-up after 9 months demonstrated ongoing use of the stimulator and continuing relief.

### Case 3

A 40-year-old male physician presented with itching, paresthesias, and when symptoms were severe, pain medial to the right scapula of 20 years duration, status post a thoracotomy performed while a child. The ipsilateral thoracotomy scar ran across the expected course of the long thoracic nerve. He reported no intercostal pain, and his NP symptoms were located several inches medial to the posterior margin of the thoracotomy scar. Past treatments included topiramate and over-the-counter analgesics. He reported 70% relief of symptoms within the first two weeks of using EMS to the serratus anterior muscle and continues daily use as of September 2009. He continues to use the stimulator intermittently after 9 months of follow-up.

### Case 4

A 34-year-old female presented with pain and pruritus medial to the right scapula, status post cervical fusions of C4-5 and C5-6 in 2006 due to degenerative joint disease. She also presented with neural foraminal stenosis in C6-7. Electrodiagnostic studies were consistent with dysfunction of C5, C6, and C7. Nonetheless, she denied radicular symptoms into the arms. Past treatments included gabapentin, desipramine, and multiple opioids. Stimulation of the serratus anterior muscle began in fall 2007. As of April 2009 she uses the stimulation for 15 minutes a day but is only intermittently compliant. When she discontinues stimulation the pain gets steadily worse prompting her to reinitiate stimulation, and continue until improvement of symptoms once again leads to discontinuation. Follow-up at 20 months following initiation of serratus stimulation revealed the patient to be using the stimulator intermittently with continued benefit.

## Discussion of findings and implications for etiologies of NP

The effectiveness of EMS, applied directly to the serratus anterior, in providing significant pain relief supports our conclusion that notalgia paresthetica can be caused by long thoracic nerve injury and consequent serratus anterior dysfunction. These findings are the first to identify the association of long thoracic nerve and serratus anterior dysfunction as a possible cause for NP, with EMS as a possible treatment. Serratus dysfunction following long thoracic nerve injury and the resulting loss of scapular protraction is well understood to lead to scapular winging. Although scapular winging was not clinically apparent in these cases, subtle shoulder girdle asymmetry was appreciated following knowledge of the diagnosis. We hypothesize that even in the absence of obvious winging, loss of protraction may result in subtle retraction of the scapula. This in turn may generate either traction or compression of the cutaneous medial branches of the thoracic dorsal primary rami of spinal nerves as they course to the skin through muscles attached to the non-protracted scapula such as the rhomboid and trapezius. Traction on these nerves would be expected to refer pain to the area between the scapula and the spine, the region of symptoms in NP.

This hypothesis would explain 1) the finding of long thoracic nerve and serratus anterior dysfunction in patients presenting with pain medial to the scapula; 2) the finding that the pain was exacerbated by activities with the arm flexed in front of the body, which loads the serratus; 3) the loss of temperature sensation in the area of pain medial to the scapular edge; 4) analgesia medial to the scapula in response to muscle stimulation of the much more lateral serratus; and 5) previous reports of the efficacy of anti-neuropathic pain medications such as gabapentin [18] and oxcarbazepine [19].

We suspect that EMS contracts the denervated serratus which is over-stretched under the load of the arm in the chronic absence of physiologic contraction. The intermittent contraction associated with the direct electrical muscle stimulation results in a durable but ultimately reversible shortening of these overstretched muscle fibers leading to a serratus muscle of shorter length, more closely approximating its normal resting length. This shorter serratus muscle, while still unable to contract physiologically, may nonetheless hold the scapula in a more anatomically correct position compared to the completely stretched, unstimulated serratus seen following denervation. Further work is needed to confirm or refute these speculations.

Our results are consistent with a case of NP and comorbid scapular winging reported in 2004, in which NP naturally resolved several months before scapular winging [20]. Interestingly, in that case scapular winging preceded the onset of NP by several months. In light of our findings, this suggests that both extent and duration of injury to the long thoracic nerve are important factors in the pathology of NP. Our report suggesting the efficacy of electrical serratus stimulation extends these observations, and implies that serratus dysfunction is causally related to the pain, and not coincidental to it.

Although our reports focus on long thoracic nerve and consequent serratus anterior dysfunction as an etiology for the clinical syndrome of NP, it is possible that serratus dysfunction is sufficient but not necessary. We have seen pain of similar presentation among those with high thoracic disk disease, and in two patients with severe scoliosis in whom the scoliosis appeared to result in impingement of the dorsal primary rami between two adjacent transverse processes. Our experiences, along with radiographic findings by others [5], also support spinal pathology as an alternative etiology of notalgia paresthetica.

Additionally, while serratus anterior dysfunction may lead to improper scapular stabilization, an imbalance of other stabilizers such as the trapezius or other posterior scapular stabilizers may provide the same symptoms. The authors suggest a defect in scapular stabilization is just one of a variety of causes of irritation to the medial cutaneous branches of the dorsal primary rami, yielding pain and pruritus in the region between scapula and spine. This hypothesis on the pathophysiology of NP would predict that the injury to other motor nerves stabilizing the scapula (e.g., the spinal accessory nerve) or the stabilizing muscles they innervate might lead to a similar clinical syndrome.

Our findings highlight the need to establish normal scapulothoracic stabilization in patients presenting with NP. In cases of NP with a well-defined etiology of injury to the serratus anterior, the long thoracic nerve, or the cervical roots it arises from, EMS of the serratus anterior muscle may be a promising treatment modality for notalgia paresthetica.

### On Dermatologic Symptoms

While pain and pruritus were both present in our patients, we note the lack of hyperpigmentation or other visible skin abnormalities which commonly accompany descriptions of notalgia paresthetica. It is unclear whether these skin abnormalities cause or are a result of the pain and pruritus in the symptomatic area. Immunohistochemical findings in NP have been inconclusive, with discordant conclusions on distributions of nerves in the symptomatic area [9-11]. Further work is required to better characterize the relationship between pain, pruritus, and hyperpigmentation, with knowledge that several distinct etiologies and consequent presentations of notalgia paresthetica may exist. Compared to neuropathic pain, itch is a commonly occurring, but less commonly appreciated, sequelae of nerve injury and regeneration [21]. For example, itch is a significant symptom in more than 40% of patients with chronic post herpetic neuralgia [22]. Two patients reported that their sense of itch was replaced by pain as symptoms became more severe. One patient noted that duloxetine first made the pain transform into an itch, and ultimately at a higher dose caused the sensation to resolve. Pain and itch share similar underlying physiology [13]. A subset of substance P transmitting c-fiber neurons, similar to those implicated in the pathogenesis of pain, has been associated with causing itch [23]. Stimulation of these neurons may directly lead to itch or itch may indirectly result from mast cell degranulation caused by substance P [24,25]. Nonetheless, both dermatologists and neurologists may fail to recognize the neuropathic nature of itch in some patients [13]. We speculate that even among patients whose sole manifestation of NP is itch, unlike those in this case series, serratus anterior or long thoracic nerve dysfunction may be to blame. Hyperpigmentation, occasionally noted in NP, may be a direct consequence of neurologic dysfunction with neurogenic release of substance P in the skin which, in addition to causing itch [23-26], also causes proliferation of keratinocytes, arterial smooth muscle cells, and fibroblasts [27]. Alternatively, the neurologic dysfunction may result in hyperpigmentation indirectly due to associated scratching, heat, or other stimuli applied to the dysesthetic skin. We did not measure pruritus in a formal way before or following treatment and so the efficacy in specifically reducing itch is not known. Future work will be of much greater value and reliability if it includes formal measurement of itch severity before and following treatment, which might include a Visual Analog Score (VAS) or another scale of symptom severity.

### On EMS versus TENS

It is possible that analgesia from electrical muscle stimulation is a result of transcutaneous electrical nerve stimulation (TENS). A study investigating TENS for relief of NP symptoms where TENS was applied to the symptomatic area medial to the scapula found roughly 30% improvement in pruritus over two weeks [28], highlighting the need to account for TENS-induced analgesia in any randomized blinded trial by including a TENS control arm. We feel it is unlikely that the analgesia from electrical nerve stimulation provided here was due to a TENS-like effect for three reasons: 1) the area of stimulation on the serratus, on the lateral side of the scapula in the axilla and ventral to the lateral border of the latissimus dorsi (Figure 2), was far removed from the area of pain; 2) pain relief was not apparent during stimulation, relief followed after several days of the treatment, and persisted for several days with only intermittent stimulation and, perhaps most convincingly, 3) several patients required fine adjustment in the placement of the stimulating pads to elicit contraction of the serratus anterior rather than the latissimus dorsi. This minute adjustment in pad placing, moving further away from the site of pain as the pads were shifted anteriorly to avoid the latissimus, made the difference between ineffective and effective stimulation. If the effect were due to a TENS-like effect or a placebo effect, the efficacy of the intervention would not be expected to differ so dramatically based on fine adjustments of pad placement far lateral to the site of pain.

### Future work

We describe a novel and well-defined etiology of long thoracic nerve injury with consequent serratus anterior dysfunction in notalgia paresthetica, and EMS of the serratus anterior as a long-acting and effective treatment conferring the advantage of a once-daily treatment regimen. Future work might expand on this case series by: 1) conducting randomized blinded trials of serratus stimulation for NP; 2) conducting case-control studies among patients with NP to evaluate the association of NP and long thoracic nerve injury; 3) performing quantitative sensory testing (QST) to document the presence of nerve injury in the area of pain among patients with NP and potential QST improvements after treatment; and 4) exploring if loss of scapular stabilization by other means, e.g. injury to the dorsal scapular nerve and spinal accessory nerve, leads to a similar clinical syndrome with respect to NP. We also note that future studies of notalgia paresthetica would benefit immensely from a multidisciplinary approach integrating the expertise of dermatologists, neurologists, orthopedic surgeons, and pain management specialists.

## Conclusion

Notalgia paresthetica is a poorly-understood condition in which patients present with pain with or without pruritus and paresthesias in an area lateral to the spine and medial to the scapula. To date, there have been various proposed etiologies of NP and a multitude of different treatment approaches which have met with varying degrees of success. We describe a novel and well-defined etiology of long thoracic nerve injury with consequent serratus anterior dysfunction in notalgia paresthetica, and EMS of the serratus anterior as a long-acting and effective treatment conferring the advantage of a once-daily treatment regimen. The authors hypothesize that abnormal scapulothoracic stabilization creates traction or compression of the cutaneous medial branches of the thoracic dorsal primary rami. This then gives rise to the symptoms of this syndrome. EMS of the serratus anterior muscle is a possible treatment for NP that deserves further study.

## Competing interests

The authors declare that they have no competing interests.

## Authors' contributions

AG conducted all electrodiagnostic studies and treated patients clinically. IRC treated patients clinically. MB and SM helped with manuscript preparation, literature review, and background context for the manuscript. SM additionally provided material resources. CKW led the literature review and prepared the manuscript. All authors critically read and approved the final manuscript.
